# Building coherence and synergy among global health initiatives

**DOI:** 10.1186/s12961-015-0062-3

**Published:** 2015-12-09

**Authors:** Fabio Zicker, Miriam Faid, John Reeder, Garry Aslanyan

**Affiliations:** Center for Technological Development in Health (CDTS), Oswaldo Cruz Foundation (Fiocruz), Av Brasil 4036, 8th floor, room 814, 21040-361 Rio de Janeiro, Brazil; Special Programme for Research Training in Tropical Disease (TDR), World Health Organization, 1211 Geneva, Switzerland

**Keywords:** Global health initiatives, International health, Neglected tropical diseases, Research and development

## Abstract

**Background:**

The fast growth of global health initiatives (GHIs) has raised concerns regarding achievement of coherence and synergy among distinct, complementary and sometimes competing activities. Herein, we propose an approach to compare GHIs with regard to their main purpose and operational aspects, using the Special Programme for Research and Training in Tropical Diseases (TDR/WHO) as a case study. The overall goal is to identify synergies and optimize efforts to provide solutions to reduce the burden of diseases.

**Methods:**

Twenty-six long-established GHIs were identified from among initiatives previously associated/partnered with TDR/WHO. All GHIs had working streams that would benefit from linking to the capacity building or implementation research focus of TDR. Individual profiles were created using a common template to collect information on relevant parameters. For analytical purposes, GHIs were simultaneously clustered in five and eight groups according to their ‘intended outcome’ and ‘operational framework’, respectively. A set of specific questions was defined to assess coherence/alignment against a TDR reference profile by attributing a score, which was subsequently averaged per GHI cluster. GHI alignment scores for intended outcome were plotted against scores for operational framework; based on the analysis of coherence/alignment with TDR functions and operations, a risk level (high, medium or low) of engagement was attributed to each GHI.

**Results:**

The process allowed a bi-dimensional ranking of GHIs with regards to how adequately they fit with or match TDR features and perspectives. Overall, more consistence was observed with regard to the GHIs’ main goals and expected outcomes than with their operational aspects, reflecting the diversity of GHI business models. Analysis of coherence indicated an increasing common trend for enhancing the engagement of developing country stakeholders, building research capacity and optimization of knowledge management platforms in support of improved access to healthcare.

**Conclusions:**

The process used offers a broader approach that could be adapted by other GHIs to build coherence and synergy with peer organizations and helps highlight the potential contribution of each GHI in the new era of sustainable development goals. Emerging opportunities and new trends suggest that engagement between GHIs should be selective and tailored to ensure efficient collaborations.

**Electronic supplementary material:**

The online version of this article (doi:10.1186/s12961-015-0062-3) contains supplementary material, which is available to authorized users.

## Background

The current dynamic and diverse global health scenario exhibits a multitude of new actors, strategies and approaches that offer new opportunities for engagement and synergy [1–3]. Some global health initiatives (GHIs) were established in response to the Millennium Development Goal priorities, mobilizing substantial new resources for health outside of traditional development assistance. However, most GHIs operate on results-driven business-like models, guided by objectives to develop or scale up access to selected treatments, services or products in health. Some initiatives focus on specific diseases, health conditions, or integrated healthcare for vulnerable populations in low- and middle-income countries [4], and operate by procedures that appeal to and connect multiple stakeholders.

GHIs, however, often differ in scope, scale and operating mechanisms in the fulfilment of the abovementioned functions. While some GHIs work as financing instruments, most initiatives also focus on advocacy, coordination, technical support, and in research and product development. Collectively, they open up a health development space and engage an increasing number of new actors and roles in global health for the benefit of poor populations [5].

One of the pioneer initiatives established in 1974 was the Special Programme of Research and Training in Tropical Diseases (TDR), co-sponsored by UNDP, UNICEF, and World Bank, and hosted by WHO. Over the years, TDR has progressively shifted its strategic emphasis from supporting basic biomedical research and product development toward applied field research and implementation/operational research, addressing issues related to access and scaling-up of interventions in a range of poverty-related diseases affecting neglected populations. Research capacity strengthening has remained a strong strategic pillar during this transition [6, 7]. Through its close working relationship with a variety of stakeholders, and in consultation with disease endemic countries and communities, TDR has continuously reassessed where and how it could make a difference [8]. Current core working areas of TDR are capacity building, knowledge management, implementation research, and research in support of neglected tropical disease control.

The evolving and expanding landscape posed the pressing question of how TDR could best position itself on the current global health scenario to optimize its impact on public health. In addition, question emerged as to how to anticipate the new landscape in the wake of the coming United Nations Sustainable Development Goals, in which health is considered as a precondition of a broader agenda. As part of TDR’s review of its potential strategic partnerships, a methodology was developed to assess coherence and synergies among GHIs, with respect to their ‘intended outcome’ and ‘operational framework’.

This paper offers a systematic review of key GHIs, looking particularly at how they relate and complement TDR, to assist in better-informed strategic choices. While the purpose of this review was to help guide TDR’s choices of scientific, technical and advocacy partnerships, it offers a broader approach that could be adapted by other GHIs to build coherence and synergy.

## Methods

### Selection of GHIs

Twenty-six long-established GHIs were identified by the authors from among all initiatives associated/partnered with TDR or WHO. All GHIs have working streams that would benefit from linking to the capacity building or implementation research focus of TDR.

The selected GHIs have worldwide activities covering a broad range of public health issues relevant to TDR, and the largest overall budget. Other GHIs not fulfilling these criteria, those which no longer reflect the core working areas of TDR, or those with insufficient publically available information were excluded.

The sample included a broad and diverse representation of initiatives, including those with a national or multinational scope, and of a public, private, for-profit, or not-for-profit and provider or implementer nature (see below). Due to the selection process, not all current and past partners of TDR were included, but the selection encompasses a wide representation of different types of GHIs.

#### Selected GHIs and acronyms (in alphabetical order)

1. The Bill & Melinda Gates Foundation (BMGF)
2. The Clinton Health Access Initiative (CHAI)
3. The Cochrane Collaboration (Cochrane)
4. Council on Health Research for Development (COHRED)
5. GHI hosted by the UK Department for International Development (DFID)
6. Drugs for Neglected Disease Initiative (DNDi)
7. European and Developing Countries Clinical Trials Partnership (EDCTP)
8. Fogarty International Center - National Institutes of Health (FIC)
9. Foundation for Innovative New Diagnostics (FIND)
10. Gavi, The Vaccine Alliance (Gavi)
11. The Global Fund to Fight AIDS, Tuberculosis and Malaria (GFTAM)
12. Global Funding of Innovation for Neglected Diseases (G-Finder)
13. Drug donation programmes in GlaxoSmithKline plc. (GSK)
14. The EU Framework Programme for Research and Innovation (Horizon 2020)
15. International Development Research Centre, Canada (IDRC)
16. The Institute for Health Metrics and Evaluation (IHME)
17. Medicines for Malaria Venture (MMV)
18. Drug donation programmes in Novartis International AG (Novartis)
19. The President’s Malaria Initiative (PMI)
20. The Rockefeller Foundation (RF)
21. Roll Back Malaria (RBM)
22. Stop TB Partnership (Stop TB)
23. International Union Against Tuberculosis and Lung Disease (The Union)
24. Innovative Financing for HIV, Malaria, TB (UNITAID)
25. United States Agency for International Development (USAID)
26. The Wellcome Trust (WT)

### GHI profiles

Individual GHI summary profiles were created using a common template for collecting publically available information on key parameters, referring to their purpose, as well as functional and programmatic set-up. The information collected included mandate, objectives, key guiding principles/objectives, founding partners, business model, priorities, decision-making body, prioritization processes, decision-making processes for financial or technical support, diseases and technologies covered, modalities of direct and indirect support, beneficiary geographical coverage, capacity building activities, knowledge management, translation of research into policy, work outreach, and business trend.

#### Clustering of GHIs

Given the diversity identified, and for better analytical purposes, two groups of GHI clusters were formed based on the profile created for each organization according to the GHIs ‘intended outcome’ – describing what the GHI seeks to achieve, and ‘operational framework’ – reflecting the GHI business model, policy, process and activities.

The following concepts were adopted:

1. Intended outcome sets out and defines what the GHI seeks to achieve. It serves as a roadmap and helps clarify the ‘raison d’être’ of the initiative, also by means of strategizing on how to define targets and achieve intermediary and final objectives. For this paper, the intended outcome was obtained from the GHIs information on its mandate, mission and priorities. The following GHI clusters were defined based on their intended outcome (a description of each cluster is available in the Additional file 1 – Description of GHI clusters).
  Access to products and services for health
  Knowledge management
  Product development
  Research and innovation
  Research and capacity development
2. Operational framework refers to the GHI’s business model, as well as to its policy, process, and activities. It helps put the purpose of the initiative into practice and guides the GHI on how it is being run. For the purposes herein, the operational framework was elaborated through the information collected on mandate, mission, decision-making processes at policy and strategy levels, and funding mechanisms. The following GHI clusters were defined based on their operational framework (a description of each cluster is available in Additional file 1 – Description of GHI clusters).

- Data platform
- GHI as foreign aid
- Innovative financing mechanism
- Drug donation within pharmaceutical company
- Philanthropic funding
- Public-private product research and development
- Scientific organization or programme
- Stakeholders coordination and support platform

### Assessing coherence and alignment

To assess coherence and alignment against TDR’s form and function, a set of specific questions was formulated covering five broad areas (Table 1). Intendent outcome was mapped through questions related to GHI overall objective and target. Operational framework was mapped through questions related to the GHI processes, areas of work, funding and outlook. A TDR reference profile was established for each question and was used for direct comparison of GHIs (Additional file 2 – TDR Reference profile).

**Table 1:** **Assessment questions on coherence and alignment with the Special Programme of Research and Training in Tropical Diseases (TDR)**

Overall objectives	
Mandate	Is the mandate of the GHI compatible with TDR’s mandate?
Principles/values	Are the guiding principles/values compatible with TDR?
Priorities	Are the priorities relevant for TDR and/or reflect trending priorities in global health, such as system approaches, preventions, universal health coverage and sustainable development goals?
Nature/scope of membership	Do the nature and scope of membership conflict with TDR’s interests? Does the GHI operate through partnerships and networks?
Inclusiveness	Is there a balanced representation between high- and low/middle-income countries?
Target	
Disease and technologies	Is the diseases and technology profile/portfolio compatible with TDR’s current portfolio and possible expansion?
Beneficiary geographical area	Are the beneficiaries of support compatible with TDR’s focus?
Beneficiary eligibility	To what extent would the beneficiaries reinforce/expand the current scope of TDR?
Harmonization	Are there attempts to harmonize process and activities with partners?
Processes	
Decision-making (policy and strategy)	Is the policy and strategy decision-making process consistent with TDR’s principles?
Prioritization (programme level)	Is the decision-making process at the programmatic level consistent with TDR?
Decision-making for grants	Is the decision-making process at the project level consistent with TDR?
Business model	Is there any aspect of the business model that could restrict TDR from engaging?
Type of support	Do the funding mechanisms conflict with TDR? Can the funding mechanisms add value to TDR?
Areas of work	
Capacity building	Would the range of capacity building activities add a value to TDR’s current approaches? If so, what kind of added value?
Knowledge management	Would the range of knowledge management add a value to TDR’s current approaches? If so, what kind of added value?
Translational research	Is the research and funding covering translational research? And if so, any evidence of success?
Funding and outlook	
Global Health Initiative (GHI) outreach	Would engagement with the GHI add another value for strengthening TDR’s influence in global health (e.g. agenda setting, networking, etc.)?
Funding	Would the engagement with the GHI offer the prospect of additional funding for TDR?
Business trend	Is there a risk for TDR to engage with the GHI? (e.g. sustainability, reputation costs)?

### Scoring

A 1–3 scale was used to assess the level of individual GHI coherence/alignment against the established TDR profile in relation to each question in Table 1. The individual comparison allowed a score of 3 = full coherence/alignment, 2 = partial, and 1 = minimum coherence/alignment. The score was attributed through consensus between two of the authors (FZ and MF), and involved a third author (GA) in case of discordance. Total scores were calculated for both individual and GHI clusters as a summary measure of how adequately they fit or match TDR’s characteristics.

A broad TDR subjective risk level for engagement was attributed to each GHI (as low, medium, high) considering the potential conflict between mandates, interference with governance and current alliances, conflict of interest between partners, potential issues of ownership and accountability, departure from WHO interest and TDR’s priorities.

## Results

The report offers a strategic approach to assess GHIs, both individually and within the functional clusters, reviewing key priority areas and added value. The qualitative assessment involved the following steps: (1) assessment and scoring of individual GHIs against the TDR reference profile for the first nine questions in Table 1, which relate to intended outcome; (2) assessment and scoring of individual GHIs against the TDR reference profile for the subsequent 11 questions in Table 1, which relate to operational framework; (3) summarizing two total scores per GHI and an average for each cluster (max score = 27 for nine questions on intended outcome and max score = 33 for 11 questions on operational framework); (4) attribute a TDR engagement risk group (high, medium or low); and (5) plot total scores for outcome versus framework for each GHI.

Table 2 shows how each particular GHI was classified/clustered according to its intended outcome and operational framework (see Additional file 1 for descriptions of clusters).

**Table 2:**
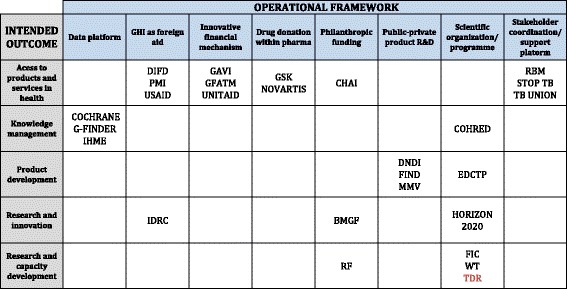
**Global Health Initiatives (GHIs) classified by intended outcome and operational framework, respectively**

GHIs full names are provided in Table 1.

Twelve GHIs were assigned to the group in which the intended outcome was to improve access to products and health services, although they operate through different approaches, such as direct technical and financial support to programme planning and implementation, drug donation, or as a coordination platform of the multiple stakeholders. Most of these GHIs are closer to public health action and have an important interaction with country level stakeholders.

Three out of the four GHIs in the knowledge management group operate mainly as data platforms, consolidating and analysing information from multiple sources. Three out of the four product development initiatives reviewed are structured as public-private partnerships, and the fourth (EDCTP) as a country partnership initiative of the European Union. All four interact with a broad range of research organizations.

The two GHI clusters with which TDR shares a strong mandate affinity are ‘research and innovation’ and ‘research and capacity development’. The three GHIs in the research and innovation group have quite distinct setting and operational frameworks (IDRC, BMGF and Horizon 2020). Three GHIs share TDR’s intended outcome on research and capacity development (RF, FIC and WT), with FIC and WT being clustered as scientific organization/programme and RF operating through a philanthropic framework.

Other ways of clustering the GHIs reviewed in this report, such as by target population, geographic or disease focus, type of support, and primary role as funding or implementing organization, could have been displayed differently to highlight alternative interfaces. However, the two parameters selected – ‘intended outcome’ and ‘operational framework’ – were the most appropriate to assess TDR positioning and potential collaboration.

**Figure 1:**
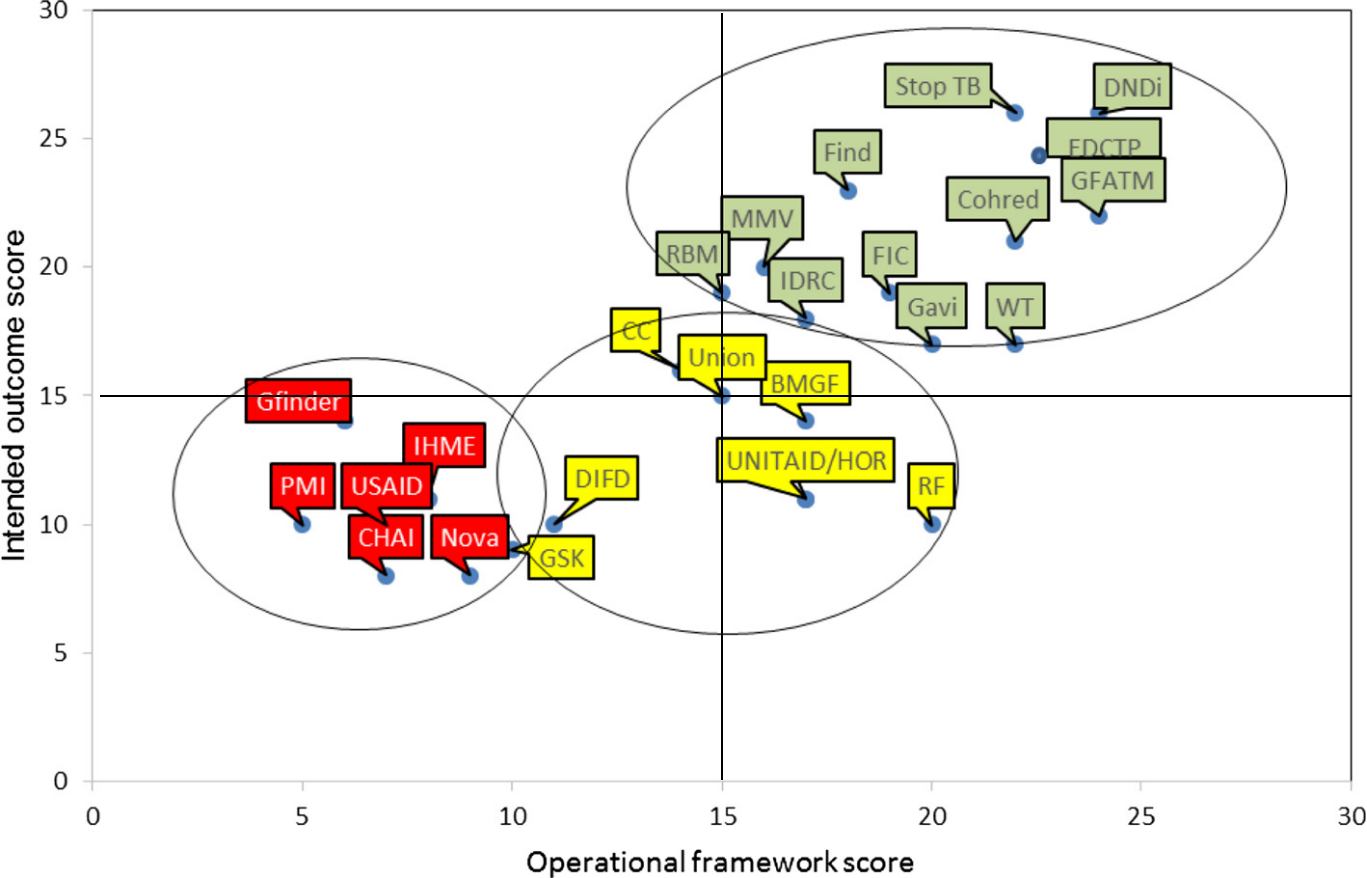
**Alignment/coherence total scores for intended outcome versus operational framework in relation to the Special Programme for Research and Training in Tropical Diseases (TDR).** Global Health Initiatives’ full names are provided in Table 1.

Figure 1 plots two summary total alignment scores for each GHI. Scores for intended outcome are plotted against scores for operational framework. The scores indicate how well the GHI matched a TDR reference profile. Additional file 3 provides individual GHI and cluster-based total scores.

In addition, the three levels of increasing risk were colour coded. This allowed for a preliminary visualization of the GHIs with regard to their potential strategic engagement with TDR.

The best fit for coherence and alignment is represented by GHIs located in the top right corner and the relatively less fitting initiatives would be in the bottom left corner. It is notable that most of the GHIs with best alignment are the ones with which TDR has more recent work collaboration. The highest risk for engagement was assigned to GHIs with the strongest independent governance and autonomy in which TDR would possibly have more operational constraints. Most of them are plotted in the bottom part of the graphic.

**Figure 2:**
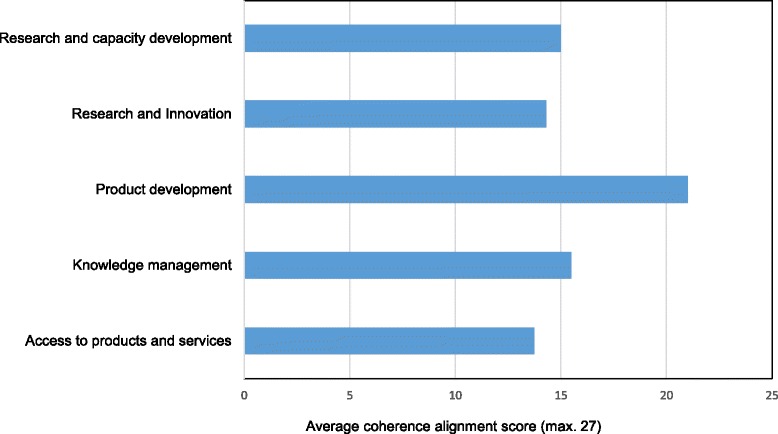
**Average coherence/alignment scores for the intended outcome cluster.**

**Figure 3:**
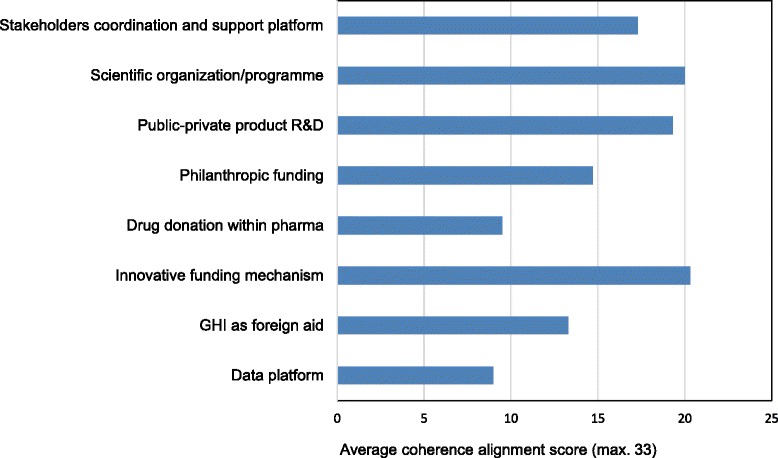
**Average coherence/alignment scores for the operational framework cluster.**

Figures 2 and 3 present the average coherence/alignment scores for clusters of intended outcome and operational framework, respectively. GHIs tend to align more in terms of proposed intended outcome than by operational framework. Product development GHIs were shown to have the most identification with TDR in the expected outcome cluster. Figure 3 indicates an inconsistent alignment among the eight GHI groups with regard to operational framework, which reflects the diversity of their business models.

## Discussion

This review comes at a particularly important time with respect to the post-2015 development agenda debate that will shape global health governance, including the role and activities of GHIs [9]. The approach used herein reveals areas of overlapping mandates and actions among GHIs, highlighting the need for more coherence, synergy and advocacy for a global health research agenda.

The methodology used for reviewing the GHIs revealed interfaces and opportunities for TDR to either reinforce its engagement or initiate new collaborations with the selected GHIs. No attempt was made to describe past and current engagements of TDR with the GHIs reviewed in this report, in order to allow for an objective assessment across all selected initiatives. The result, however, was consistent in showing stronger alignment for those GHIs with which TDR currently enjoys productive engagement.

Concerning risk for engagement, it needs to be highlighted that even long-term and reliable supporters carry a risk when there is limited influence or control of the GHIs on their own decision-making process and sustainability.

A possible limitation of the analytical approach used herein is the process of selecting the initiatives to be included in the review. The inclusion criteria should be adapted to the needs of the specific GHI carrying out the mapping exercise of its best position in the global landscape. It is hoped that critical self-review by GHIs with regards to peer organizations would promote more coherence and synergetic collaborations.

The analysis of coherence highlighted the following notable trends shared by a number of initiatives:

- Overall interest in supporting research and capacity development for improved healthcare access and implementation at country level [10].
- Optimization of the use of knowledge management platforms to assist in agenda setting and evidence-based practice [11].
- Commitment to support research capacity development as a component of large ongoing health research and innovation initiatives [12].
- Promotion and application of health system research in support of equity, and better access to products and health services [13].
- Reinforcement of the crucial role of developing countries to promote outcome research in light of the increased attention to Universal Health Coverage [14].

Given the fast-paced global health environment, GHIs should pay close attention and continuously assess emerging opportunities and new trends. Strategic engagement should thus be selective and tailored in a way that ensures efficient collaborative frameworks and long-term goals in order to minimize risks and reduce transaction costs and unwanted spin-offs.

While new GHIs will likely emerge in response to the new global development architecture, many of the currently operating initiatives will also have to reassess how their mandate and activities align with the Sustainable Development Goals to ensure relevance and impact on the newly set global health priorities.

## Conclusion

Improving health in the new era of Sustainable Development Goals will require leadership from all stakeholders. GHIs may play a unique role ensuring coherence and synergies across their activities, thus increasing the impact when delivering their activities and influencing global health in innovative and sustainable ways.

This review provides strategic information to facilitate TDR in reassessing its potential to efficiently perform in an increasingly complex global governance setting. It also highlights the diversity of features among the selected GHIs. The analytical framework used indicates possible ways forward and stimulates the development of alternative analytical approaches.

## Additional files

Additional file 1:
**Description of Global Health Initiative (GHI) clusters reviewed in the paper.** (DOC 144 kb) Additional file 2:
**Special Programme of Research and Training in Tropical Diseases (TDR) reference profile used for scoring Global Health Initiatives’ coherence and alignment.** TDR responses to questions in Table 1 to build a reference profile. (PDF 294 kb) Additional file 3:
**Individual Global Health Initiative (GHI) and cluster-based total coherence/alignment scores.** The table presents individual and cluster-based scores for GHI coherence/alignment in relation to the Special Programme of Research and Training in Tropical Diseases (TDR) reference profile. (PDF 260 kb)

## Abbreviations

GHI: Global Health Initiative
TDR: Special Programme for Research and Training in Tropical Diseases
