# Supplementary material for: Building coherence and synergy among global health initiatives
Source: Health Res Policy Syst. 2015 Dec 9;13:75. doi: 10.1186/s12961-015-0062-3 (PMC4675017; doi:10.1186/s12961-015-0062-3)
Supplement: Additional file 1: — Description of Global Health Initiative (GHI) clusters reviewed in the paper. (DOC 144 kb) [file 12961_2015_62_MOESM1_ESM.doc]

## Additional file 1. Description of the GHIs clusters

## By Intended Outcome

**Access to products and services for health**

The main purpose of these GHIs is to provide, improve or broaden access to new or underused interventions or comprehensive health care packages. Emphasis is thus usually placed on the implementation of validated preventive, therapeutic, diagnostic tools or strategies with the overarching objective of reducing the burden of disease. Most of the initiatives aim at strengthening health systems, reinforcing disease control strategies, improvement of programme efficiency, providing drugs, vaccine and supplies, in addition to supporting and conducting training to health workers. Activities can also include technical assistance and financial support for the development of national strategic plans, or the provision and/or distribution of drugs at reduced prices. Their respective mandates focus on single or a set of selected diseases of which most are disproportionately prevalent in low-and middle income countries, including HIV/AIDS, tuberculosis, malaria, TB and vaccine-preventable diseases. The targeted geographic area is headed by Africa, Asia, South America and the Caribbean, but may at times also include the Middle East and Eastern Europe.

**Knowledge management**

These GHIs seek to improve the health of people worldwide by providing high-quality, evidence-base, relevant and accessible data and related information on different fields, including population health, health determinants, the performance of health systems, disease trends, or performance of interventions, among others. The overarching goal is to make better-informed decisions in research, advocacy, policy, and practice in health. Knowledge can be produced, managed and provided in different forms, such as via data bases, reports, analytical reviews, visualization of research & development dynamics and networks, through systematic reviews or other synthesized research evidence. While some GHIs generate, manage and provide data, other GHIs serve as platforms, providing meta or aggregate data, or virtual space for members to bring together, manage and provide their knowledge. The beneficiaries of knowledge management initiatives for global health include researchers, funders, policy-makers, and any organization seeking evidence-based knowledge. Different capacity building activities are also offered, through methodology trainings, policy workshops, online learning courses, or skills-based learning tools and training resources.

**Product development**

GHIs in this group include the public-private product development partnerships aiming to develop new therapeutic and diagnostic tools or to optimize the ones available. These GHIs are mainly driven by short-term strategies, that are characterized by a focus on specific interventions and which can be delivered during a time-frame of several years, but many of these GHIs also pursue mid-to long-term intentions, according to which they seek to develop next-generation therapies. Support provided by these GHIs includes needs assessments activities, seed or core funding, technology transfer, administrative support, as well as business and development support. Capacity building activities include renovations of clinics, primary health care units and health posts, as well as the renovation, re-equipment and strengthening of clinical laboratories as needed for clinical development. Online or on-the ground training of health service personnel may also be provided and usually have an emphasis on providing expertise in clinical trial methodology, good clinical practices and research ethics, patient treatment and evaluation, accurate diagnosis, or clinical follow-up.

**Research and innovation**

These GHIs offer support to specific or cross-disciplinary research and innovation proposals on communicable or non-communicable diseases, future and emerging health trends and technologies, research infrastructure development, as well as topics including basic research, health and wellbeing, demography, or health care delivery. Further support can also include the development of research governance structures and policy frameworks for health research, priority setting of health and other related national priorities, or policy development. Priority is given to low- and middle-income countries health issues, and here frequently on supporting individuals or organizations working in research and policy environments. Grants or calls that seek to attract grant submissions from industrialized countries have a stronger focus on tackling broader challenges at regional or global level. Much of the support offered by these GHIs aims to further translation, research to products and implementation, but also aims for research to inform and influence policy-making processes.

**Research and capacity development**

GHIs in this group are essentially committed to advance scientific knowledge and research capacity. A common focus here is to strengthen individual and research institutions or help develop international research networks. A large range of diseases, technologies and disciplines are frequently included, covering communicable and non-communicable diseases, clinical, operational or health services research, innovation across the health care pipeline, m-Health or E-Health. Key areas of research and capacity building activities vary and change according to the different priorities and funding schemes provided by these GHIs. Although frequently focusing on low-and middle-income countries, other world regions or specifically selected high-priority countries are also regularly covered, and special research and capacity strengthening initiatives often exist for Africa. While some GHIs have also started to place strategic attention on supporting emerging countries, others have actually excluded some of these from their beneficiary list of low- and middle income countries, as they are now considered as upper middle-income countries.

**By Operational Framework**

**Data platform**

GHIs that operate as data platforms usually offer different kinds of online informative data. Data is provided in different forms of presentation, aggregation and visualization to inform users about states of investment, trends, or patterns in global health. Datasets may have been produced by the GHI itself, its platform users, or represent a collection of external data sets provided by other institutions. While some data platforms are merely available on the web pages of the GHIs for consultation purposes, others may also be sent to its users or downloaded along with different software tools to allow users to replicate research methods and data. Key principles of these GHI are: making global health data easier to find, compare and interpret; promote data sharing; raising awareness of global health information; transparency of information and decision-making; scientific relevance for global health; the importance of evidence-based policy-making; increasing collaboration; impartiality; stimulating increased efficiency and investment in global health; or minimizing bias. Data platforms serve a variety of user groups, such as public and private organizations, philanthropic actors, NGOs, researchers and policy-makers, health practitioners and media services.

**GHIs as foreign aid**

GHIs can also form part of a government’s foreign aid structure, with global health generally figuring as one focus area among many others, which together aim at ending extreme poverty and to promote the development of societies. While its main agent is the national government, these GHIs may also often include a network of different partners for its operational activities. Key guiding principles usually focus on the strengthening of its country’s presence and influence in foreign affairs, in combination with its domestic socio-economic development and other national or global interests. Some of them have specific attention to improving access to disease-specific treatment including: HIV/AIDS, tuberculosis, malaria, maternal and child health, health systems, neglected tropical diseases, as well as cross-cutting health areas. Direct support channeled to partner countries consists of grants, funds for research or for programme implementation, cooperation agreements, but also the provision of commodities or technical assistance. Capacity building activities may also occur as a form of assistance, both for the partner countries or contracted partner organizations.

**Innovative financing mechanism**

This group of GHIs represents institutional mechanisms that attract new sources of investment intended to fill financial gaps and provide sustainable, predictable or additional funding for public health. Revenues can take different forms, including products and services, new charges of fees collected from different sectors, or new sources of public and private investment. They either operate as fundraising mechanisms – often at the global level and aligned with global priorities – or they deliver financial solutions on the ground, at country or community-level. Innovative financing mechanisms in global health are often realized through public-private alliances or partnership, commonly including government donors from both developed and developing countries, international organizations, the private sector, non-governmental and philanthropic actors, civil society, and health research institutes. The mechanisms can operate to positively influence financing flows for specific diseases, but predominantly seek to significantly influence market dynamics to reduce prices and thereby increase the availability of drugs, vaccines, diagnostic tools and other supplies. As a result, most innovative financing mechanisms are commodity-driven, operating by use of market power and market dynamics, but they may also seek to directly influence producer-consumer relationships.

**Drug donation within pharmaceutical companies**

Besides their for-profit core focus, a significant number of pharmaceutical transnational companies also actively engage in corporate social responsibilities with a focus on selected global health challenges. To date, drug donation has occurred in form of single pledges, but also on the basis of multi-year commitments signed between the company and the respective international organization or country. A number of these activities are realized through collaboration with other public and private stakeholders. The target group is commonly the least developed countries, having WHO as the intermediate facilitator to reach the most needed populations. GHIs can also promote capacity building activities, with a focus on supporting the training of healthcare workers in developing countries, but also extending to activities that seek to help build scientific, regulatory, medical and manufacturing capacities so as to attract and absorb new technologies from the private sector, including the transfer of know-how.

**Philanthropic funding**

GHIs existing in form of philanthropy are pursued by sponsors such as corporate and family foundations, private funds, or collaborations with other not-for-profit organizations. The general operating framework is characterized by a business-like set-up and philosophy and often makes use of management techniques that resemble the private sector, with an emphasis on efficiency, performance goals and (social) investment returns. Philanthropic funding can be of grant-making or direct operating character. These GHIs include in their philanthropic portfolio a broad range of global health issues and disease-specific matters such as HIV, tuberculosis, malaria, and neglected infectious diseases, but in some cases also include pneumonia, enteric & diarrheal diseases. Depending on the specific grant schemes of these GHIs, other diseases and disease-relevant technologies, or discovery & translational sciences are also covered. The geographical coverage of beneficiaries is worldwide, though an emphasis and interest is frequently placed on low and middle-income countries. Different forms of capacity building may exist as support component of the GHI’s funding schemes. As their funding sources are separate from public budgets, philanthropic funding is considered by some analysts as being able to apply more risk-taking attitudes than traditional funding mechanisms and as result, it is argued to be potentially more innovative on the way and amount of the allocated funds.

**Public-private product R&D**

These organizations usually operate to bridge existing gaps in research and development on neglected portfolios of drugs, vaccines, and medical diagnostics. They initiate and coordinate respective R&D programmes or projects in collaboration with selected partners from the international research community, the public and private sectors and also clinical centers, making use of the distinct comparative advantage of each stakeholder and providing incentives for these to join. Important components of this operating framework include a patients-driven focus, the intention to break the link between the costs and incentives of traditional R&D engagement from the price of products, a commitment to make innovative scientific knowledge affordable and accessible, as well as to ensure sustainability of research & development products, and the possibility of technology transfer. A number of product R&D organizations also promotes access to biological reagents, provides support for appropriate clinical and regulatory studies or other forms of knowledge management, tools and equipment that serve as incentives for stakeholders to engage in new product R&D. In return, the partners commit to providing their services and products to targeted markets under agreed upon conditions.

**Scientific organization/programme**

These GHIs seek to stimulate and improve excellence in knowledge, research and innovation by providing support for basic, clinical, applied research and training. Flexible funding systems exist for different scientific strands and thematic areas. Grantees are usually individuals, national or international research collaboration groups, both of specific disciplinary or multidisciplinary characters. Scientific organizations or programmes may also facilitate scientific exchanges among investigators and across scientific projects, provide training opportunities for improving scientific investigations, and support communication outreach of supported scientific projects by sharing information and results on their web pages and other outlets. Some are also directly involved in scientific production or help providing technical and scientific expertise and tools at individual, institutional, as well as health and innovation systems levels. In global health, these operate as charity organizations, governmental programmes, or non-governmental organizations, either at national regional or global level. Most global health initiatives operate as not-for-profit driven organizations or programmes. If they operate as governmental components, they are predominantly set up as a means to strengthen their respective country’s research and innovation system and thereby maximize quality and leadership, or support the country’s domestic and external policies.

**Stakeholder coordination and support platform**

GHIs clustered in this group convene a wide range and number of stakeholders relevant for the respective global health area. They may operate as autonomous bodies or be hosted by larger organizations, including the World Health Organization. Most of its operations are set out for mobilizing actions and resources from its stakeholders, facilitating dialogue, forging consensus, and coordinating activities among these so as to avoid duplication and fragmentation of activities and resources. Members are commonly expected to agree upon a specific set of priorities, goals and strategies. Stakeholders can include governments of developing and developed countries, bilateral and multilateral development partners, the private sector, non-governmental-, and community-based organizations, foundation, research, academic and technical institutions, funding agencies, foundations, as well as individuals. Together, these activities may also seek to stimulate and test out new ideas and solutions to global health challenges and engage in advocacy worldwide.
