# Supplementary material for: Building coherence and synergy among global health initiatives
Source: Health Res Policy Syst. 2015 Dec 9;13:75. doi: 10.1186/s12961-015-0062-3 (PMC4675017; doi:10.1186/s12961-015-0062-3)
Supplement: Additional file 2: — Special Programme of Research and Training in Tropical Diseases (TDR) reference profile used for scoring Global Health Initiatives’ coherence and alignment. TDR responses to questions in Table 1 to build a reference profile. (PDF 294 kb) [file 12961_2015_62_MOESM2_ESM.pdf]

Additional file 2 - TDR reference profile used for scoring GHIs' coherence and alignment

Assessment questions (from Annex 1)		TDR reference profile
**Overall objectives**		
Mandate	Is the mandate of the GHI compatible with TDR´s mandate?	Supports research-driven, innovation for health impact projects and initiatives
Principles/ values	Are the guiding principles/values compatible with TDR?	Impact, quality, inclusiveness, multi-stakeholder approach
Priorities	Are the priorities relevant for TDR and/or reflect trending priorities in global health, such as system approaches, preventions, universal health coverage and sustainable development goals?	Implementation research, intervention strategies for infectious diseases of poverty
Nature/ scope of membership	Does the nature and scope of membership conflict with TDR´s interests? Does the GHI operate through partnerships and networks?	UN-based co-sponsored organization with donors and beneficiaries representation on its policy and decision-making boards
Inclusiveness	Is there a balanced representation between high and low-middle-level income countries?	Balanced at all levels of representation
**Target**		
Disease and technologies	Is the diseases and technology profile/portfolio compatible with TDR´s current portfolio and possible expansion?	Selected NTDs + malaria, TB and technologies related to IR
Beneficiary geographical area	Are the beneficiaries of support compatible with TDR´s focus?	Individuals and institutions in high-burden DEC
Beneficiary eligibility	To what extent would the beneficiaries reinforce/expand the current scope of TDR?	Trend forgeographic expansion in terms of number and type of beneficiaries
Harmonization	Are there attempts to harmonize process and activities with partners?	Efforts for multi-stakeholders harmonization (eg. ECWG)

**Processes**		
Decision-making (policy & strategy)	Is the policy- and strategy decision-making process consistent with TDR´s principles?	Broad stakeholder representation for decision-making
Prioritization (programme level)	Is the decision-making process at the programmatic level consistent with TDR?	Principles of transparency and engagement of the relevant stakeholders, especially beneficiaries
Decision-making for grants	Is the decision-making process at the project level consistent with TDR?	Independent, peer-review decision-making and transparency
Business model	Is there any aspect of the business model that could restrict TDR from engaging?	Not-for-profit, based on partnerships and networks, leadership in DECs
Type of support	Do the funding mechanisms conflict with TDR? Can the funding mechanisms add value to TDR?	Mix of proactive and investigator-initiated proposals for support
**Areas of work**		
Capacity building	Would the range of capacity building activities add a value to TDR´s current approaches? If so, what kind of added value?	Training and institutional development, networks and regional capacity
Knowledge Management	Would the range of knowledge management add a value to TDR´s current approaches? If so, what kind of added value?	KM related to priority setting, harmonization, systematic /thematic reviews
Translational research	Is the research and funding covering translational research? And if so, any evidence of success?	Implementation research, operational research
**Funding and outlook**		
GHI outreach	Would the engagement with the GHI add another value for strengthening TDR´s influence in global health (e.g. agenda setting, networking, etc.)?	CEWG-related, convening power for priority-setting in GH
Funding	Would the engagement with the GHI offer the prospect of additional funding for TDR?	70% of current funding from a small number of traditional donors
Business trend	Is there a risk for TDR to engage with the GHI? (e.g. sustainability, reputation costs)?	Partners are expected to share ownership, add on original mandate, respect values and principles, improve visibility and perception
