# Supplementary material for: Building coherence and synergy among global health initiatives
Source: Health Res Policy Syst. 2015 Dec 9;13:75. doi: 10.1186/s12961-015-0062-3 (PMC4675017; doi:10.1186/s12961-015-0062-3)
Supplement: Additional file 3: — Individual Global Health Initiative (GHI) and cluster-based total coherence/alignment scores. The table presents individual and cluster-based scores for GHI coherence/alignment in relation to the Special Programme of Research and Training in Tropical Diseases (TDR) reference profile. (PDF 260 kb) [file 12961_2015_62_MOESM3_ESM.pdf]

Additional file 3. Individual and cluster-based total scores for Global Health Initiative (GHIs) coherence and alignment with TDR*

GHIs clustered by intended outcome **	Total score (max=27)
**Access to products and services**	
CHAI	8
DFID	10
GAVI	17
GFATM	22
PMI	10
Roll Back Malaria	19
Stop TB	26
The Union	15
UNITAID	11
USAID	10
GSK	9
Novartis	8
Cluster average	14
**Knowledge management**	
Cochrane Collaboration	16
COHRED	21
G-FINDER	14
IHME	11
Cluster average	16
**Product development**	
DNDi	21
FIND	21
MMV	19
EDCTP	24
Cluster average	21
**Research & Innovation**	
Horizon 2020	11
IDRC	18
Bill & Melinda Gates Foundation	14
Cluster average	14
**Research and capacity building**	
FIC	19
Rockefeller Foundation	10
Wellcome Trust	17
Cluster average	15

GHIs clustered by operational framework	Total score (max=33)
**Data platform**	
Cochrane Collaboration	13
G-FINDER	6
IHME	8
Cluster average	9
**GHI as foreign aid**	
DFID	11
PMI	5
USAID	7
IDRC	17
Cluster average	13
**Innovative financing mechanism**	
GAVI	20
GFATM	24
UNITAID	17
Cluster average	20
**Drug donation with pharma**	
GSK	10
Novartis	9
Cluster average	10
**Philanthropic funding**	
Bill& Melinda Gates Foundation	17
CHAI	7
Rockefeller Foundation	20
Cluster average	15
**Public-private R&D**	
DNDi	24
FIND	18
MMV	16
Cluster average	19
**Scientific organization/programmes**	
COHRED	22
Horizon 2020	17
FIC	19
Wellcome Trust	22
EDCTP	22
Cluster average	20
**Stakeholders coordination support**	
Roll Back Malaria	15
Stop TB	22
The Union	15
Cluster average	17

* Scores based on comparison of individual GHIs characteristics against a TDR profile for question in Annex 1

** GHIs full names are provided in Box 1
